# Epidemiological and Molecular Characterization of an Invasive Group A Streptococcus *emm*32.2 Outbreak

**DOI:** 10.1128/JCM.00191-17

**Published:** 2017-05-23

**Authors:** Jennifer E. Cornick, Anmol M. Kiran, Roberto Vivancos, Jon Van Aartsen, Jenny Clarke, Edward Bevan, Mansoor Alsahag, Maaike Alaearts, Laura Bricio Moreno, Howard F. Jenkinson, Angela H. Nobbs, James Anson, Aras Kadioglu, Neil French, Dean B. Everett

**Affiliations:** a Malawi Liverpool Wellcome Trust Clinical Research Programme, Blantyre, Malawi; b Institute of Infection & Global Health, University of Liverpool, Liverpool, United Kingdom; c Field Epidemiology Service, Public Health England, London, United Kingdom; d NIHR Health Protection Research Unit in Emerging and Zoonotic Infections, University of Liverpool, Liverpool, United Kingdom; e Heart of England NHS Foundation Trust, Birmingham Heartlands Hospital, Birmingham, United Kingdom; f School of Oral and Dental Sciences, University of Bristol, Bristol, United Kingdom; g Liverpool Clinical Laboratories, Royal Liverpool & Broadgreen University Hospitals Trust, Liverpool, United Kingdom

**Keywords:** iGAS, streptococci, Streptococcus pyogenes, epidemiological data, whole-genome sequencing, phylogeny, accessory genome, comparative genomics, virulence factors, antibiotic resistance, molecular epidemiology

## Abstract

An *emm*32.2 invasive group A streptococcus (iGAS) outbreak occurred in Liverpool from January 2010 to September 2012. This genotype had not previously been identified in Liverpool, but was responsible for 32% (14/44) of all iGAS cases reported during this time period. We performed a case-case comparison of *emm*32.2 iGAS cases with non-*emm*32.2 control iGAS cases identified in the Liverpool population over the same time period to assess patient risk factors for *emm*32.2 iGAS infection. The *emm*32.2 iGAS cases were confined to the adult population. We show that homelessness, intravenous drug use, and alcohol abuse predisposed patients to *emm*32.2 iGAS disease; however, no obvious epidemiological linkage between the patients with *emm*32.2 iGAS could be identified. Comparative whole-genome sequencing analysis of *emm*32.2 iGAS and non-*emm*32.2 control isolates was also performed to identify pathogen factors which might have driven the outbreak. We identified 19 genes, five of which had previously been implicated in virulence, which were present in all of the *emm*32.2 iGAS isolates but not present in any of the non-*emm*32.2 control isolates. We report that a novel *emm*32.2 genotype emerged in Liverpool in 2010 and identified a specific subset of genes, which could have allowed this novel *emm*32.2 genotype to persist in a disadvantaged population in the region over a 3-year period.

## INTRODUCTION

Lancefield group A beta-hemolytic streptococcus (GAS), also called Streptococcus pyogenes, is a frequent commensal of the human oropharynx and also an important human pathogen, responsible for ∼750 million infections annually (1). Clinical disease ranges in severity from mild pharyngitis and impetigo to life-threatening necrotizing fasciitis (2). GAS disease has been described in all human populations, although certain groups appear particularly susceptible (3). Other notable features of GAS epidemiology include the propensity for long-term fluctuations in disease rates, such as the recent substantial increase in scarlet fever in the United Kingdom in 2013/2014 (4), and the association with outbreaks. GAS outbreaks are strongly associated with long-term-care or institutional facilities (5–8) and occur less often within whole communities (9–11). The introduction of new *emm* genotypes (12, 13) or the acquisition of prophages harboring virulence genes by previously circulating GAS are the primary mechanisms underpinning these outbreaks (14–16).

The antiphagocytic M surface protein, encoded by the *emm* gene, is a major virulence factor and also an epidemiological marker, which is used to type GAS isolates (3); over 230 *emm* types have been described to date (4). In addition to the M protein, virulence is determined by adhesion proteins, by toxin production, and by the absence or presence of a capsule, and is influenced by host factors (5). GAS is also typed based on multilocus sequence typing (MLST); 651 MLST variants have been reported to date (17).

Here, we describe the emergence and persistence of a novel GAS *emm* type, *emm*32.2, in Liverpool, Merseyside, United Kingdom. The *emm*32.2 genotype was unusual in that it showed an association with repeated invasive GAS (iGAS) cases over a period of 3 years, almost wholly confined to Liverpool and to an adult population without a clear epidemiological linkage. We utilized comparative genomic analyses of the *emm*32.2 outbreak isolates with other invasive and noninvasive isolates, recovered from individuals within the same community and same time frame, to identify genomic features unique to the *emm*32.2 type which may have driven the ability of this *emm* type to emerge and persist within the population.

## RESULTS

### Emergence of *emm*32.2 iGAS in Liverpool.

**FIG 1:**
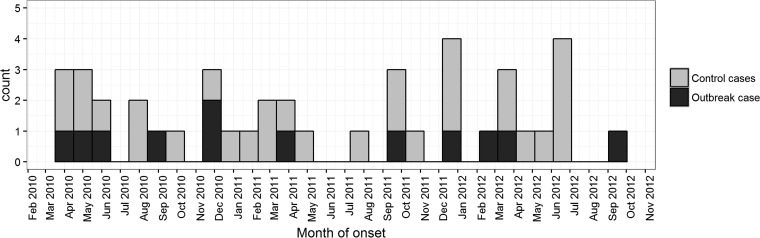
Histogram plot of all iGAS cases in Liverpool from January 2010 to December 2012 reported to the Respiratory and Vaccine Preventable Bacteria Reference Unit of Public Health England. Over this time period, 14 outbreak *emm*32.2 outbreak iGAS cases were reported.

Fourteen cases of *emm*32.2 iGAS were recorded in Liverpool (*n* = 12) and the wider Merseyside area (*n* = 2) between January 2010 and September 2012, all confined to the adult population with a median age of 47 years (range, 18 to 65 years). Over the study period, *emm*32.2 accounted for 32% (14/44) of all iGAS cases reported in Liverpool. Prior to January 2010, *emm*32.2 iGAS had not been reported in Liverpool and only three *emm*32.2 iGAS cases had ever been reported in the United Kingdom to the Respiratory and Vaccine Preventable Bacteria Reference Unit (RVPBRU) previously; one in Birmingham and two in Glasgow (R. Vivancos, unpublished data). No *emm*32.2 cases have subsequently been reported to the RVPBRU since September 2012. Figure 1 shows a histogram of the iGAS cases during the study period.

### Case-case comparison.

The case fatality rate associated with *emm*32.2 iGAS outbreak cases did not differ from the case fatality rate of the non-*emm*32.2 iGAS cases reported from the Liverpool population during the same time period (25% versus 10%, respectively; *P* = 0.374). We performed a case-case comparison study to investigate risk factors associated with the *emm*32.2 iGAS outbreak cases (Table 1). Only *emm*32.2 iGAS cases (*n* = 12) from Liverpool residents were included in the epidemiological investigations; *emm*32.2 iGAS cases (*n* = 2) from the two wider Merseyside area residents were not included as these patients could not be contacted for follow-up. The outbreak cases were confined to the adult population; of the 30 control cases, 25 (83%) were reported in the adult population, the remaining five (17%) were reported in children. However, the outbreak cases did not differ significantly in age or sex from the control patients with non-*emm*32.2 iGAS. Furthermore, patient interview data collected by Public Health England (PHE) suggested that the frequency of recent travel was low in both the cases and the controls with no statistically significant difference of travel between these two groups (8% versus 0%, respectively; *P* = 2.0). Of interest, however, outbreak cases were more likely to occur in the homeless (30% versus 0%, respectively; odds ratio [OR], 1.59 × 10^8^; *P* < 0.001), intravenous drug users (50% versus 5%, respectively; odds ratio [OR], 10; *P* = 0.009) and alcohol abusers (42% versus 5%, respectively; OR, 14; *P* = 0.002) relative to the control cases. A multivariate logistic regression model identified both homelessness and alcohol abuse as independent factors (Akaike information criteria [AIC], 37.7), yet these factors only explain about 50% of the outbreak cases. The geographical distribution of outbreak cases showed areas of higher concentration, whereas that of controls was more disperse; however, further interview of cases found no common epidemiological link between the outbreak cases.

**TABLE 1:** Case-case comparison of clinical and demographic characteristics associated with iGAS cases^*a*^

Factor	No. of cases (%)		Odds ratio (confidence interval)	*P* value
	Outbreak (*n* = 12)	Control (*n* = 30)		
Age (years)				0.132
<1	0 (0)	1 (3)	1	
1–9	0 (0)	2 (7)	1 (0–∞)	
10–19	1 (8)^*b*^	2 (7)	2.13 × 10^7^ (0–∞)	
20–29	1 (8)	3 (10)	1.42 × 10^7^ (0–∞)	
30–39	1 (8)	1 (3)	4.25 × 10^7^ (0–∞)	
40–49	4 (34)	3 (10)	5.67 × 10^7^ (0–∞)	
50–59	4 (34)	4 (13)	4.25 × 10^7^ (0–∞)	
≥60	1 (8)	14 (47)	3.04 × 10^6^ (0–∞)	
Male sex	7 (58)	15 (50)	1.4 (0.36–5.41)	0.624
Travel	1 (8)	0 (0)	2.45 × 10^14^ (0–∞)	0.202
Deceased	3 (25)	4 (13)	2.17 (0.4–11.6)	0.374
Pregnant	0 (0)	3 (10)	0 (0–∞)	0.146
IVDU^*c*^	6 (50)	2 (7)	14 (2.25–87.02)	0.002
Homeless	4 (34)	0 (0)	1.59 × 10^8^ (0–∞)	<0.001
Alcohol	5 (42)	2 (7)	10 (1.59–62.78)	0.009
Influenza-like illness	0 (0)	2 (7)	0 (0–∞)	0.239

a Outbreak (*emm*32.2) and nonoutbreak control cases belonging to multiple *emm* types that were reported in Liverpool, United Kingdom, between January 2010 and December 2012.

b This case was in an 18-year-old.

c IVDU, intravenous drug use.

### *In silico* genotyping.

**FIG 2:**
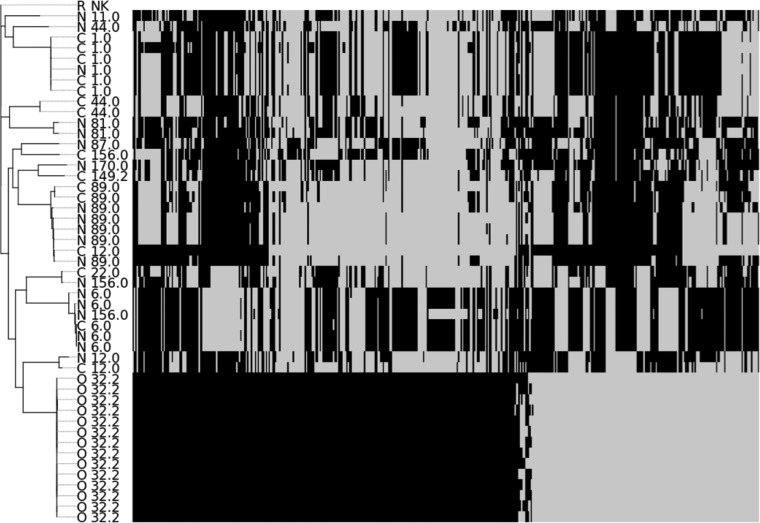
Maximum likelihood phylogeny of the 48 GAS isolates based on core genome SNPs. Each branch is annotated with the isolate source followed by the in *silico emm* type. R, the GAS reference sequence Streptococcus pyogenes HKU16; C, control iGAS cases; N, control noninvasive isolates; and O, outbreak *emm*32.2 iGAS cases. (Right) The absence (gray) or presence (black) of accessory genes in each strain in no particular order. An expanded version of this figure, showing the gene names is presented in Table S1 in the supplemental material.

We sequenced 48 GAS isolates, including all 14 *emm*32.2 outbreak isolates, 15 non-*emm*32.2 iGAS isolates, and 19 noninvasive isolates that were not part of the outbreak. Following whole-genome sequencing, *in silico emm* typing and multilocus sequence typing were performed on each isolate (Fig. 2; see also Table S1 in the supplemental material). Fourteen different sequence types (ST) were identified in the data set. In concordance with the RVPBRU molecular typing results, *in silico* typing confirmed all of the outbreak iGAS cases as *emm*32.2. The *emm*32.2 isolates were further characterized as ST386. There are only five previous reports of ST386 in the pubmlst.org database, all from iGAS cases in the Czech Republic between 2003 and 2008 (http://pubmlst.org/spyogenes/). The nonoutbreak isolates consisted of 12 different *emm* types and 12 different STs.

### Virulence genes unique to the outbreak isolates.

Following orthologous clustering of genes identified in the annotated genomes, the genes from all isolates were divided into two groups: orthologous genes shared by all isolates (core genes) and those not shared by all isolates (accessory genes). We identified 5,971 orthologous clusters in the data set, of which 1,294 represented core genes. A phylogeny based on single nucleotide polymorphism (SNP) differences between the concatenated core genes is presented in Fig. 2. Within the phylogeny, the emergent *emm*32.2 outbreak isolates form a distinct clade, clearly separated from the nonoutbreak isolates. The phylogeny indicates that the *emm*32.2 isolates are genetically distinct from the other isolates circulating within the same population during the same time period. To identify genes that may have driven the outbreak, we compared the accessory gene content of the outbreak versus the control isolates. We identified 19 accessory genes that were present in all of the outbreak isolates but completely absent in the rest of the data set (Table 2 and Fig. 2; see also Table S1). Five of the accessory genes unique to *emm*32.2 could be assigned a definitive function and have previously been implicated in bacterial virulence, including an Mga-like regulatory protein, an Myr positive regulator, LepA, trypsin-resistant surface protein T6, and a hyaluronidase (HylP) (Table 2).

**TABLE 2:** Predicted functions of 19 accessory genes unique to *emm*32.2 outbreak isolates^*a*^

Cluster no.^*b*^	Representative sequence ID^*c*^	Most significant BLAST match^*c*^	Query coverage (%)	E value	Identity (%)	Accession no.
183	101008_01052	Endodeoxyribonuclease/NUMOD4 motif protein	100	1.00E−131	100	WP_011017973.1
1734	101008_00230	GTP-binding protein LepA (SipA)	100	4.00E−76	100	WP_002984850.1
1882	101008_01097	HNH endonuclease	100	3.00E−86	100	WP_032460877.1
1847	101008_01057	Hyaluronidase protein (HylP)	100	0	99	WP_023079488.1
1842	101008_01051	Hypothetical protein	100	1.00E−136	100	WP_002991560.1
1925	101008_01398	Hypothetical protein	98	4.00E−50	100	EFM32866.1
1921	101008_01377	M protein, *trans*-acting regulator (Mga)	95	4.00E−160	99	KGE57367.1
1922	101008_01378	M protein, Myr positive regulator	100	8.00E−38	100	KGE57368.1
1919	101008_01354	Short-chain fatty acid transporter domain protein	100	5.00E−92	100	WP_021340353.1
1917	101008_01349	Trypsin-resistant surface protein T6	100	0	100	WP_038433629.1
1923	101008_01380	Protein precursor	100	0	85	WP_063629327.1
1914	101008_01346	—^*d*^	—	—	—	—
1920	101008_01376	—	—	—	—	—
1885	101008_01100	—	—	—	—	—
1881	101008_01096	—	—	—	—	—
1785	101008_00610	—	—	—	—	—
1784	101008_00609	—	—	—	—	—
1840	101008_01049	—	—	—	—	—
1908	101008_01284	—	—	—	—	—

a Predicted functions based on a comparison of the amino acid sequence to the NCBI BLAST database.

b Accessory genome clusters containing the nucleotide sequences and translated amino acid sequences of the *emm*32.2 unique genes are available at https://datahub.io/dataset/liverpool-gas.

c Representative sequence identifier (ID) represents the specific amino acid sequence from each cluster that was used as an input query sequence for comparison to the NCBI database.

d —, no match was available in the NCBI BLAST database.

### Prophage acquisition.

**FIG 3:**
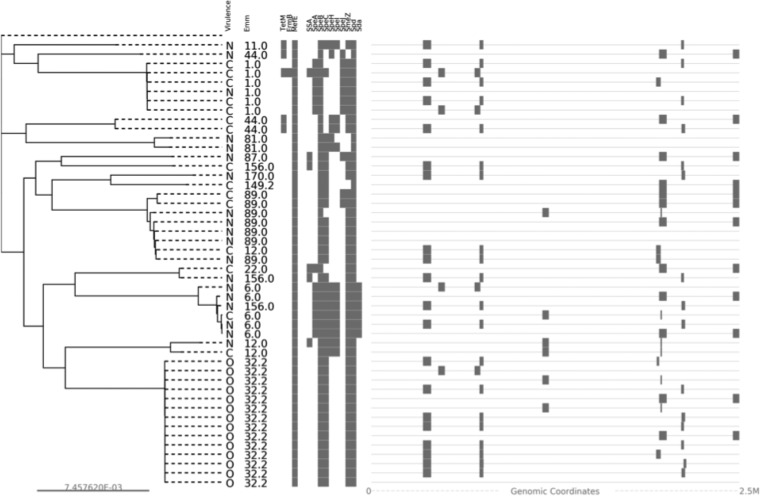
Maximum likelihood phylogeny of the 48 GAS isolates based on core genome SNPs. Each branch is annotated with isolate source followed by the in *silico emm* type. R, the GAS reference sequence Streptococcus pyogenes HKU16; C, control iGAS cases; N, control noninvasive isolates; and O, outbreak *emm*32.2 iGAS cases. (Middle) Antibiotic resistance and superantigen genes (in gray) in the genomes of the study isolates. (Right) Predicted genomic locations of putative prophage elements in each study isolate.

Previous studies have documented that the acquisition of bacteriophages carrying virulence genes has driven GAS outbreaks (14–16). Therefore, we next investigated the location of the *emm*32.2 unique genes within the assembled genomes to assess if any were associated with a bacteriophage. The study isolates encoded between one and three intact bacteriophages, ranging in size from 9,978 bp to 55,428bp. However, no bacteriophage was shared by all of, or was unique to, the *emm*32.2 isolates (Fig. 3). Furthermore, none of the *emm*32.2 unique genes corresponded to regions of the genome where bacteriophages were identified. This suggests that prophage acquisition did not drive the *emm*32.2 outbreak.

### Superantigens and antibiotic resistance.

We screened the sequenced isolates for the distribution of known GAS toxins to assess if specific superantigen profiles were unique to the *emm*32.2 isolates. Of the 22 superantigens that we screened for, none were unique to the *emm*32.2 population. All of the toxins identified in the *emm*32.2 population were also present in a large proportion of the control isolates (Fig. 3). No antibiotic susceptibility data were available from the reference laboratory; therefore, we next screened the sequenced isolates for the distribution of antibiotic resistance genes. No antibiotic resistance gene profiles were unique to the *emm*32.2 isolates relative to the nonoutbreak isolates (Fig. 3). This indicates the *emm*32.2 outbreak was not driven by the acquisition of superantigens or antibiotic resistance mechanisms.

## DISCUSSION

We have shown that an outbreak of *emm*32.2 iGAS cases in Liverpool (between January 2010 and September 2012) was confined to the adult population in a small geographical area. Whole-genome sequencing of outbreak clones is a powerful tool for characterizing the biological properties, for rapidly identifying the virulence factors, and for expanding our understanding of the pathogenesis of the organisms under investigation. Through high-resolution core and accessory genome analyses, we have demonstrated that the emergent *emm*32.2 isolates harbored a combination of 19 genes not identified in other isolates circulating in the same community during the same time frame. A subset of the 19 genes have well-characterized roles in virulence and pathogenesis, namely, genes encoding an Mga-like regulatory protein, an Myr positive regulator, LepA, trypsin-resistant surface protein T6, and a hyaluronidase (HylP). The Mga DNA binding protein has been shown to induce the expression of a number of important virulence factors (18). Natural variation leading to a 12-bp deletion within the Mga promoter has been shown to result in a significantly decreased virulence of GAS in an *in vivo* model (19). Nonsynonymous SNPs resulting in an amino acid change at position 201 of the translated Mga protein were found to have occurred multiple times in the evolutionary history of *emm*59 iGAS during an epidemic outbreak in Canada (20). The presence of an alternative Mga regulator within the outbreak isolates could lead to an increased expression of virulence factors within the outbreak isolates and may, in part, explain the increased mortality rate observed in the outbreak cases compared with that in the controls. The canonical Myr regulator protein is known to contribute to the resistance to phagocytosis through controlling the transcription of the antiphagocytic M protein. Therefore, the presence of the Myr protein in the *emm*32.2 isolates likely contributed to the emergence of the *emm*32.2 clone in the Liverpool population by allowing it to evade the host immune response (21). LepA (also known as SipA), a putative signal peptidase essential for pilus assembly, and the *tee6* gene, encoding the pilus shaft protein T6, were also unique to the *emm*32.2 isolates. The T6 protein forms part of the fibronectin-binding protein, collagen-binding protein, and trypsin-resistant antigen (FCT) type 1 pilus (22). Pilus structures are an important virulence factor in streptococci and play key roles in tissue adherence, biofilm formation, and extracellular translocation (23). Deletion of *tee6* has been shown to compromise the ability to form biofilms in the majority of the GAS isolates studied (22). The assembly of the type 1 pilus in the *emm*32.2 isolates is likely to contribute to the virulence of the outbreak isolates. Finally, unique to the *emm*32.2 isolates was the *hylP* gene encoding a hyaluronidase. Hyaluronidases are a key streptococcal virulence factor and are believed to facilitate bacterial spread within host tissues by breaking down the hyaluronic acid component of the extracellular matrix (24). Hyaluronidase has also been implicated in permitting GAS to utilize hyaluronic acids from the host and its own capsule as an energy source (25). Given that these genes have been strongly implicated in virulence and in evasion of the host response, we propose that they have driven the emergence and spread of this invasive *emm* type in susceptible individuals in this population over other isolates of GAS. Further characterization of the other genes that were unique to the *emm*32.2 isolates but could not be assigned definitive functions is needed to evaluate the specific role of these genes in GAS virulence. The identification of these novel genes not previously characterized in the isolates under investigation clearly demonstrates the ability of whole-genome sequencing to rapidly generate new research questions surrounding the factors driving the pathogenesis of outbreak clones.

We have shown that in contrast to the majority of other reported iGAS outbreaks in communities, the *emm*32.2 outbreak we report is of particular interest as it was not driven by the acquisition of prophage elements, superantigens, or multidrug resistance, but rather by the introduction of a novel *emm* type into the susceptible Liverpool population. We speculate that the complement of virulence factors that we have identified in the *emm*32.2 outbreak isolates that were not present in other iGAS isolates circulating in this population during the same time period could have allowed this *emm* type to readily disseminate and infect largely immunologically naive individuals within the population. Clearly, for *emm*32.2 to have caused iGAS disease over a 3-year period in the absence of evidence of a direct epidemiological link (i.e., person-to-person), it must have at least persisted in this population and been carried in other individuals and may have caused mucosal disease. Therefore, host factors may also play a role. We present important clinical data that show that an increased odds ratio of *emm*32.2 iGAS infection is linked to intravenous drug use, alcohol abuse, and homelessness, suggesting that the clone most readily infects the disadvantaged populations. In an outbreak of *emm*59 iGAS reported in specific regions of Canada, illegal drug use, alcohol abuse, and homelessness were also all major risk factors for *emm*59 iGAS (26). Future studies to cross reference the *emm*32.2 unique genes to *emm*59 and other iGAS genotypes known to cause community outbreaks, predominantly confined to disadvantaged populations, may establish if these genes are specific markers of “outbreak” potential. Outside the United Kingdom, the *emm*32.2 genotype has only been identified once before in 2004 in the blood of a patient with orbito cellulitis in the Czech Republic. While there are only five previous reports of ST386 in the pubmlst.org database, likewise, all of these are from iGAS cases in the Czech Republic identified between 2003 and 2008. Within the United Kingdom, *emm32.2* has only previously been identified in isolated cases in Glasgow and Birmingham. It cannot be established from this analysis how this *emm* type was introduced into the Liverpool population, although recent travel did not dictate an increased odds ratio of *emm*32.2 iGAS.

In summary, we have documented a community outbreak of iGAS in Liverpool that was caused by the iGAS genotype *emm*32.2. Comparative genomic analysis of the outbreak isolates to nonoutbreak isolates has given us a high-resolution view of the GAS population that is not afforded by conventional molecular typing methods and has revealed that the ability of the *emm*32.2 genotype to disseminate in the Liverpool population was dictated by the presence of 19 genes unique to this outbreak genotype, including five genes with well-defined roles in virulence. We propose this subset of genes allowed *emm*32.2 iGAS to persist in a disadvantaged population over a 3-year period.

## MATERIALS AND METHODS

### Epidemiological investigation.

Invasive GAS (iGAS) infection is defined as the isolation of GAS from normally sterile sites (e.g., blood) or the isolation of GAS from nonsterile sites (e.g., wounds) in the presence of streptococcal toxic shock syndrome or in the presence of necrotizing fasciitis. In the United Kingdom, iGAS is a reportable disease (27), with all causative isolates sent to the RVPBRU of PHE for molecular typing. In September 2012, a notification of an iGAS case following hospital admission was reported in an adult male in Liverpool, Merseyside. The causative isolate typed as *emm*32.2 using RVPBRU molecular protocols (https://www.cdc.gov/streplab/m-proteingene-typing.html). At the time, it was noted that a further two cases of *emm*32.2 iGAS had been diagnosed at the same hospital in Liverpool in 2012. An investigation was initiated by PHE to determine whether there were any common exposures in these cases or the potential for nosocomial infection. All three cases had been community acquired and admitted with symptoms of sepsis. A review of the RVPBRU typing database identified that additional *emm*32.2 iGAS cases had been reported in the same community since January 2010. From January 2010 to September 2012, the RVPBRU confirmed a total of 14 cases of *emm*32.2 iGAS, including 12 residents in Liverpool and two in the wider Merseyside area. No additional *emm*32.2 iGAS cases have subsequently been reported in this region. Over the same time period, 30 non-*emm*32.2 iGAS infections were reported in Liverpool (Table 1). Case-case analysis was performed using data prospectively collected for the 12 *emm*32.2 iGAS outbreak cases and the 30 nonoutbreak iGAS cases reported in Liverpool residents from information supplied to the public health team (including telephone interviews with patients) and patient case note reviews. Patients infected with *emm*32.2 iGAS with illness were designated “outbreak cases,” whereas patients infected with non-*emm*32.2 iGAS were designated “controls.” Differences in demographic and clinical data between case and controls were assessed by estimating odd ratios (ORs) using logistic regression analyses. We compared the geographical distributions of outbreak cases and controls using kernel density estimates (KDE) to smooth the distributions.

### Sequencing collection.

All 14 *emm*32.2 outbreak and 15 non-*emm*32.2 iGAS isolates (randomly selected from the 30 nonoutbreak iGAS cases available) and a convenience sample of 20 noninvasive pharyngitis GAS isolates supplied by the Royal Liverpool University Hospitals Trust and Alder Hey Children's Hospital were subjected to whole-genome sequencing. The local microbiology laboratories do not routinely retain noninvasive isolates; therefore, the noninvasive isolates were actively collected pharyngitis cases diagnosed in Liverpool from June through July 2012, overlapping with the period at the end of the outbreak. Noninvasive isolates were provided with age (in years) and sex only, without other clinical details. All GAS isolates were cultured on Columbia horse blood agar (Oxoid, Basingstoke, United Kingdom) at 37°C with 5% CO_2_, and DNA was extracted for sequencing using previously described protocols (28).

### Genome sequencing and genotyping.

Whole-genome paired-end short reads (100 bp) were generated using the Illumina Genome Analyzer II (Illumina, Madison, USA) at the Centre for Genomic Research, University of Liverpool, as previously described (29). *In silico emm* typing from the short-read data were performed using a method reported by Athey et al. (30). MLST was determined using the short read sequence typing tool (SRST) (31). The Illumina sequencing statistics are available for download at http://cgr.liv.ac.uk/illum/LIMS4128_11dacd433440bef9/.

### *De novo* genome assembly and phylogenetic analysis.

*De novo* genome assembly from short-read data was performed using Velvet V1.2, with kmer size optimized through VelvetOptimiser (32). The annotation of assembled genomes was performed using PROKKA (33). The identified translated open reading frames (genes) were grouped into orthologous clusters using proteinOrtho4 (34). The sequences in each orthologous cluster were aligned with PRANK (35) and back-translated into codon alignments. Orthologous clusters that contained genes from >45 of the 48 study isolates were classed as “core genes” and concatenated to produce a core genome alignment of all study isolates. A stringent quality control process was applied to the assembled genomes; however, a relaxed core genome size of >45/48 was applied to allow for poor assembly of individual genes, which may have caused the size of the core genome to be underestimated. All orthologous clusters that contained ≤45/48 were designated “accessory genes.” A maximum likelihood phylogeny based on SNPs within the core genome alignment was reported using RAxML, with 100 random bootstrap replicates (36). Prophages were detected in the assembled genomes and analyzed using the free web tool PHAST (PHAge Search Tool) (37). Superantigens and antibiotic resistance genes were identified using the Antibiotic Resistance Identification By Assembly (ARIBA) tool (https://github.com/sanger-pathogens/ariba). A list of the antibiotic resistance genes used as the input data for ARIBA are available on the comprehensive antibiotic resistance database (CARD [38]); a list of the superantigens can be found in the report by Spaulding et al. (39). Nucleotide and translated amino acid sequence of the orthologous gene clusters generated as part of this study are available for download at https://datahub.io/dataset/liverpool-gas.

### Ethics.

Ethical approval was not required, as the study was conducted as part of a Health Protection Agency (now Public Health England) outbreak investigation.

### Accession number(s).

All of the Illumina sequence reads generated from the study isolates were deposited in the European Nucleotide Archive under the study accession number PRJEB20045. Individual accession numbers for short-read data generated from each of the study isolates are listed in Table S1 in the supplemental material.

## Supplementary Material

Supplemental material
