# Supplementary material for: Epidemiological and Molecular Characterization of an Invasive Group A Streptococcus emm32.2 Outbreak
Source: J Clin Microbiol. 2017 May 23;55(6):1837–46. doi: 10.1128/JCM.00191-17 (PMC5442540; doi:10.1128/JCM.00191-17)
Supplement: Supplemental material [file supp_55_6_1837__index.html]

Epidemiological and Molecular Characterization of an Invasive Group A Streptococcus emm32.2 Outbreak — Supplemental material 

# Epidemiological and Molecular Characterization of an Invasive Group A Streptococcus *emm*32.2 Outbreak

## Supplemental material

- Supplemental file 1 -

  Table S1 (Absence or presence of a gene from each study isolate in an accessory cluster, with accession number, *emm* type, and MLST of each isolate)

  XLSX, 1.5M
